# FGFR2 expression relates to subtype-specific tumour microenvironment (TIME) during luminal breast cancer evolution

**DOI:** 10.3389/fonc.2025.1655438

**Published:** 2025-09-12

**Authors:** Julia Sołek, Aleksandra Zielińska, Radzisław Kordek, Hanna Romańska, Marcin Braun

**Affiliations:** Department of Pathology, Chair of Oncology, Medical University of Lodz, Lodz, Poland

**Keywords:** breast cancer, ductal carcinoma in situ, invasive breast cancer, tumour immunemicroenvironment, tumour-infiltrating lymphocytes, tumour-associated macrophages, fibroblast growth factor receptors, FGFR2

## Abstract

**Background:**

Fibroblast growth factor receptor 2 (FGFR2) is an oncogenic driver in luminal breast cancer (BCa), with emerging evidence linking it to tumour immune microenvironment (TIME) modulation. While FGFR2’s role in endocrine resistance is established, its potential involvement in shaping immune infiltration—particularly in the transition from ductal carcinoma *in situ* (DCIS) to invasive ductal carcinoma (IDC)—remains underexplored.

**Methods:**

This retrospective study analysed 99 BCa specimens collected between 2004–2019. Immunohistochemistry was used to assess FGFR2 expression and immune markers (CD8, CD68, CD163, FOXP3). Clinical and pathological variables were evaluated, and immune cell densities were compared across disease stages and BCa subtypes (luminal vs. non-luminal). Correlations between FGFR2 expression and immune markers were assessed using non-parametric statistical tests.

**Results:**

Progression from DCIS to IDC was associated with increased infiltration by CD8+ T cells and CD68+ macrophages. FGFR2 expression showed differences between DCIS and IDC with an extensive DCIS component and was positively correlated with CD8+, CD163+, and FOXP3+ cell densities. The latter associations were exclusive to luminal A tumours, with no such correlations observed in non-luminal subtypes.

**Conclusions:**

FGFR2 expression in luminal A BCa correlates with markers of immunosuppressive TIME, particularly CD163+ macrophages and FOXP3+ T cells. These subtype-specific interactions suggest a synergistic role of FGFR2 and estrogen receptor signalling in immune evasion and tumour progression, warranting further mechanistic and therapeutic investigation. However, the small number of cases in certain subgroups, particularly DCIS and non-luminal tumours, limits the generalizability of these findings and warrants cautious interpretation.

## 1 Background

Intercellular communication with the tumour microenvironment (TME) mediated by the family of highly conserved transmembrane tyrosine kinase receptors (FGFR1-4), is a well-recognized mechanism of breast cancer (BCa) progression. In particular, the FGFR2 has recently emerged as a potent oncogenic driver in the luminal BCa (1, 2), and its activation by its cognate ligands, the TME-derived fibroblast growth factors (FGFs), was shown to affect the function of steroid receptors and to promote development of resistance to endocrine therapy (3–6).

There is some pre-clinical evidence to suggest that the involvement of FGFR signalling in BCa biology might not be restricted to their FGF-induced effect on BCa cells, and that modulation of FGFRs by the stimuli derived from the tumour immune microenvironment (TIME), particularly, tumour-infiltrating lymphocytes (TILs) and tumour-associated macrophages (TAMs), contributes to tumour immune evasion and invasive progression (7–9). Reported associations of FGFR expression level with several features of an immunosuppressive microenvironment, such as decrease of T-cell infiltration, enhanced Treg survival, M2-like polarization of TAMs, and downregulating MHC expression on tumour cells, seem to support this notion (7–11).

Breast cancer progression from ductal carcinoma *in situ* (DCIS) to invasive ductal carcinoma (IDC) is accompanied by dynamic changes in TIME (12). While DCIS is typically characterized by low immune infiltration, a gradual increase in immune cell presence is observed during the transition to IDC. These TIME alterations are also subtype-specific: luminal breast cancers (BCa), although generally considered less immunogenic than HER2-positive or triple-negative subtypes (13–15), demonstrate progressive immune activation during IDC progression. This suggest that this increase in immunogenicity in luminal BCa may not be incidental, but rather influenced by FGFR2-mediated modulation of TIME. In this context, FGFR2 may play an active, subtype-specific role in shaping the immune landscape during tumour evolution, contributing to the transition from DCIS to IDC even in tumours with initially low baseline immune activity.

## 2 Methods

### 2.1 Patient selection and samples

This study included 99 breast cancer (BCa) specimens collected from patients treated at the Regional Oncologic Centre of the Copernicus Memorial Hospital in Łódź and the Holy Cross Cancer Centre in Kielce, Poland, between 2004 and 2019. The cohort consisted of 27 pure DCIS cases (without an invasive component, hereafter called DCIS), including 23 luminal and 4 non-luminal subtypes, as well as 72 cases with invasive ductal carcinoma with co-existing DCIS (hereafter called IDC-DCIS), comprising 47 luminal and 25 non-luminal BCa specimens. Flow chart of patient recruitment is presented on **Supplementary Figure 1**. Clinicopathological characterization of the cohort is presented in **Supplementary Table 1**. The study was approved by the Local Ethics Committee (Approval No. KE/16/21 and RNN/284/13/KE).

### 2.2 Collection of the clinical and pathological data

For each patient, clinical data were collected, including age at diagnosis, menopausal status, type of treatment, occurrence of relapse, disease progression, and survival outcomes (date of relapse, disease progression, death, or last follow-up). The diagnosis was established according to the 2012 World Health Organization (WHO) Classification of Breast Tumours, and the disease stage was recorded.

Pathological data included nuclear grade, mitotic index, hormonal receptor status, tumour size, surgical margin status and breast cancer phenotype. Additionally, the morphological characteristics of DCIS were obtained, including histoarchitectural pattern, nuclear grade, presence of necrosis.

### 2.3 Tissue specimen, immunohistochemistry

Formalin-fixed paraffin embedded (FFPE) tissue blocks with BCa postoperative specimens were collected. Each paraffin-embedded sample was sectioned into 3.5-μm-thick slices and stained with haematoxylin and eosin (H&E) for histopathological evaluation. Immunohistochemical staining for a panel of selected biomarkers (**Supplementary Table 2**) was performed using a protocol recommended by the manufacturer.

### 2.4 Evaluation of TIL, TAM and FGFR2 expression

Morphological and semi-quantitative analysis of stromal TIICs was carried out on H&E and IHC preparations using an UltraFast Scanner (Philips) with DigiPath™ software (Xerox), following the International Guidelines on TIL Assessment in Breast Cancer. The ‘hot-spots’ was defined as areas rich in TIICs adjacent to tumour cells were selected for further phenotypical analysis, i.e. cells positive for CD4, CD8, CD68, CD163, FOXP3 were counted for each tumour in four representative areas of 0.25 mm2 under magnification of 200×. Additionally, CD4/CD8 and CD68/CD163 ratios were calculated. Counting was conducted independently by two researchers (JS and AZ) and supervised by the pathologist (MB). In case of significant disparities between the scores (20 or 5 cells/mm2 for TILs or TAMs, respectively, or difference >20% of the mean value), the case was additionally reassessed by another pathologist (HRK).

FGFR2 levels were quantified according to the semiquantitative H-score approach by two independent pathologists (JS,AZ). The data were presented in 0–300 scale resulting from multiplication of percentage of positive cells by intensity of staining: 0—no staining, 1–3—increased intensity of both cytoplasmic and membrane staining (subgroups by H-score: 0–75 for negative/weak; 76–150 for moderate; 151–225 for strong; 226–300 for very strong expression). Cases from 1st tercile of H-score were regarded as FGFR2low and cases from 2nd and 3rd terciles were classified as FGFR2high.

### 2.5 Statistical analysis

Continuous variables were presented as medians with interquartile ranges (IQR), and categorical variables as counts with percentages in parentheses. The Shapiro–Wilk test was used to assess normality of distribution. As data presented non-normal distribution, comparisons between two groups were made using the Mann–Whitney U test, and across multiple groups using the Kruskal–Wallis test with Conover–Inman *post hoc* analysis. Associations between categorical variables were assessed using Pearson’s chi-squared test. Correlations between continuous variables were evaluated using Spearman’s rank correlation coefficient. Statistical analyses were performed using Statistica version 13.1 (Dell Inc., Round Rock, TX, USA). A p-value below 0.05 was considered statistically significant.

## 3 Results

### 3.1 Histopathological and immune features of DCIS and DCIS with invasive component

Of the 99 patients included, 27 (27.3%) had DCIS, and 72 (72.7%) had IDC-DCIS. There were no significant differences between DCIS and IDC-DCIS in terms of age, grade and menopausal status (**Supplementary Table 1**). In terms of tumour size, all cases DCIS were diagnosed as pTis, whereas in the IDC-DCIS group, there was equal distribution between pT1 and pT2 (p>0.05, **Supplementary Table 1**). The estrogen receptor (ER) positivity rate did not differ significantly between the groups (p=0.0862, **Supplementary Table 1**). In contrast, HER2 protein was more frequently detectable and showed higher abundance in DCIS compared to IDC-DCIS (p<0.0001, **Supplementary Table 1**) tumours. Infiltration by total TILs and TAMs, as assessed on H&E-stained sections, was higher in IDC-DCIS compared to DCIS lesions (p=0.0001 and p=0.0002, respectively, **Table 1**). Consistently, immunohistochemical evaluation showed increased infiltration of both CD8+ T cells and CD68+ macrophages in IDC-DCIS compared to DCIS groups (p<0.0001, **Table 1**). Moreover, CD68/CD163 ratio was also increased in IDC-DCIS tumours (p=0.0004, **Table 1**). No significant differences were observed in densities of CD163+ or FOXP3+ cells (p=0.4925 and p=0.8717, respectively, **Table 1**).

**Table 1:** Characteristics of tumour-infiltrating immune cells, FGFR2 expression and ER and HER2 status in the study group.

Variable	DCIS (n=27) Median (LQ-UP)	IDC-DCIS (n=72) Median (LQ-UP)	p-value
TILs (HE)	75 (16-155)	475 (180-1555)	0.0001*
TAMs (HE)	57 (0-152)	240 (60-619)	0.0002*
CD4	75 (0-60)	240 (0-155)	0.5351
CD8	38 (0-110)	390 (70-1285)	<0.0001*
CD68	40 (0-60)	243 (70-830)	<0.0001*
CD163	0 (0-30)	0 (0-8)	0.4925
FOXP3	0 (0-50)	0 (47)	0.8717
CD4/CD8 ratio	0.25 (0.06-0.7)	0.27 (0.03-0.8)	0.8330
CD68/CD163 ratio	1.6 (1.2- 3.9)	17 (6-168)	0.0004*
FGFR2 (H-score)	60 (0-120)	40 (1-120)	0.8448

Data presented as medians, interquartile ranges in brackets, units for each variable are included in square brackets. Groups were compared using Kruskal–Wallis test. Statistically significant results are mark with asterisk.

### 3.2 Relationship between immune markers and extent of DCIS compartment

**Figure 1:**
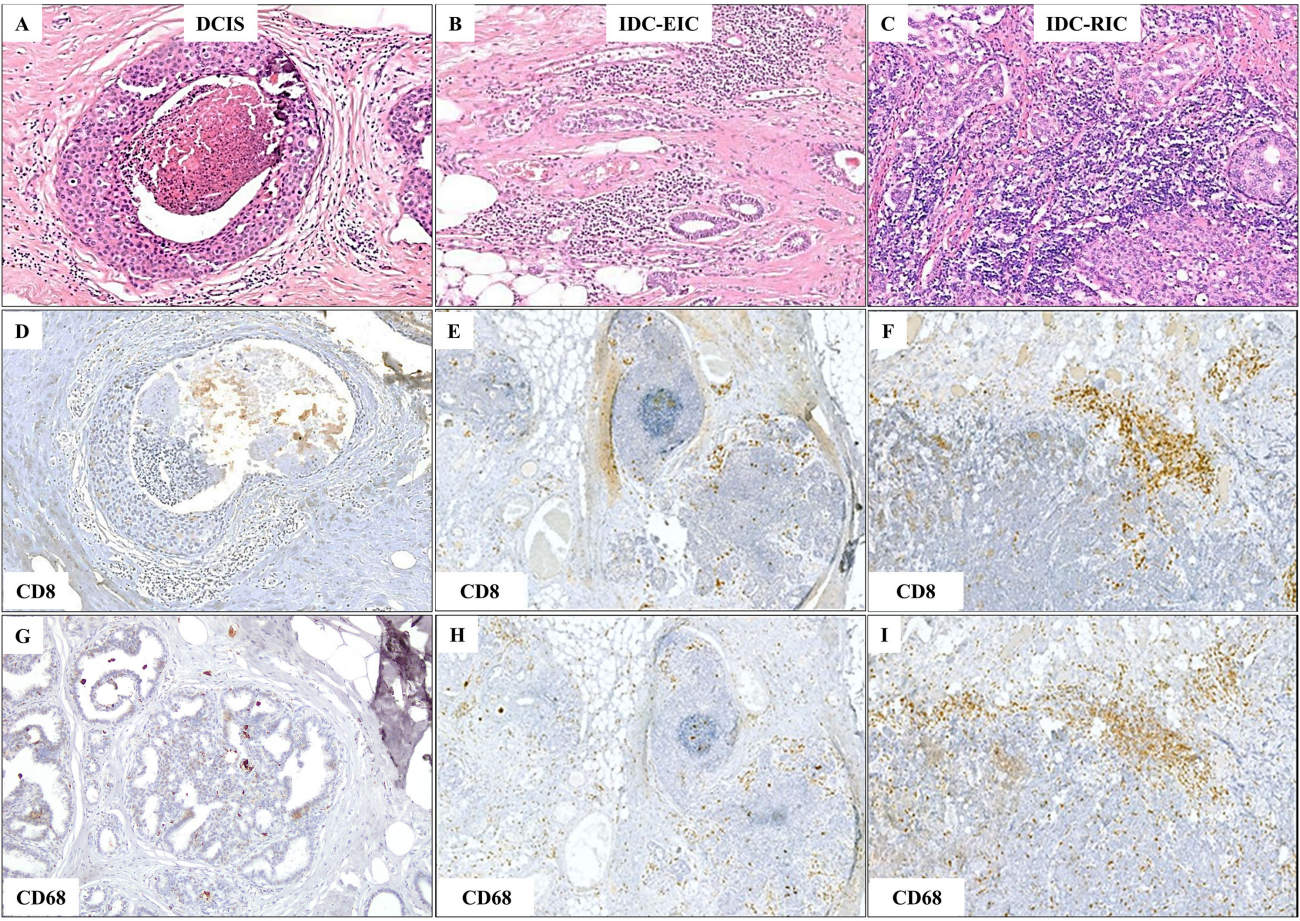
Gradual increase in TIL and TAM density observed in representative areas of DCIS, invasive ductal carcinoma with an extensive intraductal component (IDC-EIC), and IDC without EIC (IDC-RIC). **(A–C)** TILs and TAMs on H&E-stained sections in DCIS **(A)**, IDC-EIC **(B)**, and IDC-RIC **(C)**. **(D–F)** CD8 expression in DCIS **(D)**, IDC-EIC **(E)**, and IDC-RIC **(F)**. **(G–I)** CD68 expression in DCIS **(G)**, IDC-EIC **(H)**, and IDC-RIC **(I)**.

**Figure 2:**
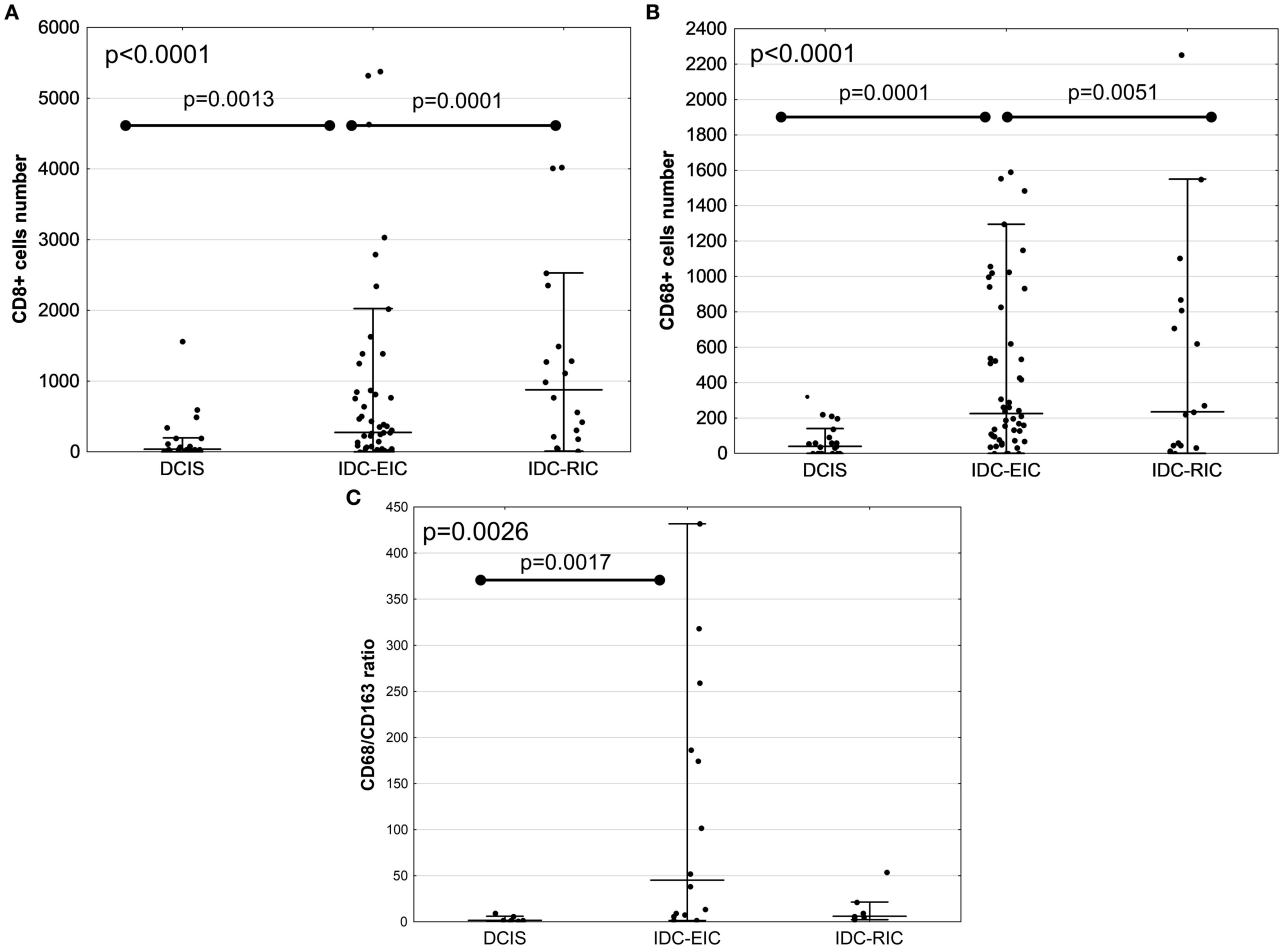
Comparison of immune marker infiltration between DCIS, IDC with EIC (IDC-EIC), and IDC without EIC (IDC-RIC). Infiltration by CD8+ T cells, CD68+ macrophages, and the CD68/CD163 ratio (**A**–**C**, respectively). Groups were compared using Kruskal–Wallis ANOVA test.

Previous studies suggest that IDC with an extensive DCIS component (extensive intraductal component- EIC) is associated with a more favourable prognosis than IDC with a less extensive DCIS involvement (restricted intraductal component – RIC), supposedly representing an intermediate stage between DCIS and fully invasive carcinoma (16). Based on the extent of DCIS component within the tumour, IDC-DCIS cases were sub-grouped into two categories: (1) IDC with EIC (IDC-EIC), defined as IDC with ≥30% DCIS (n = 48), and (2) IDC without EIC (IDC-RIC), defined as IDC with <30% DCIS (n = 16). Results showed that, when this stratification of the cohort was applied, a stepwise increase of infiltration by TILs, TAMs, CD8+ T cells, and CD68+ macrophages from DCIS via IDC-EIC to IDC-RIC was observed (AKW p < 0.0001; **Figures 1**, **2A, B**, **Supplementary Table 3**, **Supplementary Figure 2**). Interestingly, the CD68/CD163 ratio peaked in the IDC- EIC group (AKW p = 0.0026; **Figure 2C**, **Supplementary Table 3**), but neither densities of CD4+, CD163+, or FOXP3+ cells nor the CD4/CD8 ratio differed between the subsets (**Supplementary Table 3**). A similar association with a gradual increase of infiltration by TILs, TAMs, CD8, CD68 as well as the CD68/CD163 ratio was observed for tumour size (pTis →pT1→pT2). (**Supplementary Figure 3**, **Supplementary Table 4**).

### 3.3 Correlations between immune markers and FGFR2 expression

**Figure 3:**
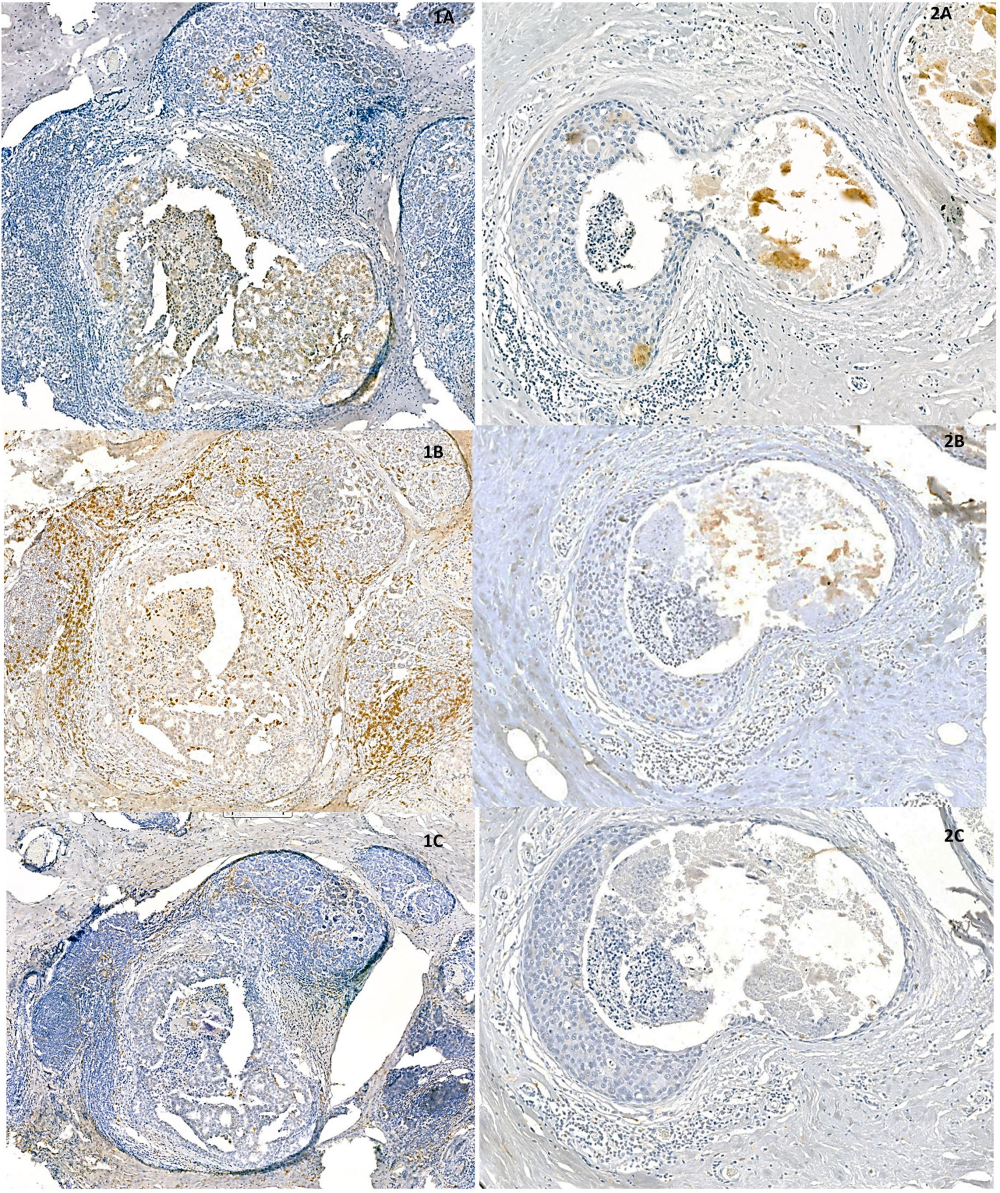
Representative areas of two, densely **(1A–1C)** and sparsely **(2A–2C)** infiltrated DCIS samples show positive correlation between expression of FGFR2 **(1A, 2A)** and densities of CD8 - positive **(1B, 2B)** and CD163 – positive cells **(1C, 2C)**.

As FGFR2 is thought to be involved in BCa progression, FGFR2 expression at specific stages of BCa evolution was investigated. FGFR2 expression in BCa cells did not differ between DCIS and IDC-DCIS tumours (p=0.8448, **Table 1**). However, in the subgroup analysis, the FGFR2 H-score was significantly higher in IDC-EIC cases compared to pure DCIS (p = 0.0154, **Supplementary Figure 4**). Next, taking into account the possible association between the FGFR2-TIME crosstalk and BCa evolution, the relationship between expression of FGFR2 on tumour cells and markers of TIME’s features was analysed in all cases (N=99) (7, 17). Although FGFR2 expression (H-score) did not correlate with total numbers of either TILs or TAMs, as measured on HE-stained sections (p=0.2067 and p=0.6086, respectively, **Supplementary Table 5**), when analysed in relation to specific phenotypes of infiltrating cells, positive correlations with densities CD8+ T-cells (p=0.0491, R=0.21), CD163+ macrophages (p=0.0161, R=0.27) and FOXP3+ T-cells (p=0.0233, R=0.25) (**Figure 3**, **Supplementary Figure 5**, **Supplementary Table 5**) were noticed. There was no association of FGFR2 with either CD4+ T-cells, CD68+ macrophages, CD68/CD163 or CD4/CD8 ratios (**Supplementary Table 5**).

### 3.4 FGFR2 correlation with immune markers is restricted to ER positive patients

Taking into account the reported oncogenic role of FGFR2 in luminal A BCa (3–5), we then evaluated the relationship between FGFR2 and immune markers in relation to the ER status, i.e. in luminal A (n=70) versus non-luminal A tumours (n=29). The data showed that the correlations between FGFR2 expression and infiltration by both CD163+ TAMs (p = 0.0027, R = 0.38) and FOXP3+ regulatory TILs (p = 0.0031, R = 0.37) were observed in patients with the luminal A subtype but not in patients with non-luminal BCa (**Table 2**, **Supplementary Figure 6**). Moreover, the correlations between CD163, FOXP3 and FGFR2 (previously found in whole group -**Supplementary Table 5**) maintained exclusively in patients with the luminal A subtype (**Table 2**). These findings suggest that the immunomodulatory role of FGFR2 supporting BCa evolution may indeed be restricted to the luminal A subtype.

**Table 2:** Correlations between FGFR2 expression (H score) and immune markers in patients with and without luminal A phenotype.

Breast cancer subtype	Variable correlated with FGFR2	R	p
Luminal A phenotype	TILs	0.13	0.3210
	TAM	0.07	0.5593
	CD4	0.02	0.8609
	CD8	0.23	0.0643
	CD68	0.14	0.2555
	CD163	0.37	0.0027*
	FOXP3	0.37	0.0031*
	CD4/CD8	-0.32	0.1287
	CD68/CD163	-0.06	0.7823
Non luminal A phenotype	TILs	0.26	0.2170
	TAM	0.19	0.3899
	CD4	0.17	0.4373
	CD8	0.28	0.1673
	CD68	0.21	0.1673
	CD163	0.17	0.4604
	FOXP3	0.96	0.6540
	CD4/CD8	-0.2493	0.3899
	CD68/CD163	-0.64	0.1730

To calculate the correlations the Spearman’s rank correlation coefficients were used. Statistically significant results are marked with asterisk.

## 4 Discussion

The present study provides an insight into a novel aspect of FGFR2 involvement in the progression of breast cancer (BCa). The analyses reveal direct correlations between FGFR2 expression and immune cell infiltration in early-stage luminal BCa specimens, that are subtype- specific and relevant particularly to estrogen receptor-positive (ER+) disease. These findings build on the established role of FGFR2 as an oncogenic driver in luminal BCa and offer new perspectives on how it may facilitate immune escape during tumour evolution.

In this context, breast cancer progression from ductal carcinoma *in situ* (DCIS) to invasive ductal carcinoma (IDC) was accompanied by a gradual increase in immune infiltration, particularly involving CD8+ T cells and CD68+ macrophages. These changes were consistent with previous reports and underline the stepwise immunological evolution of BCa (18). Our findings extend this knowledge by demonstrating that FGFR2 expression is positively correlated with markers of an immunosuppressive TIME, namely CD163+ macrophages and FOXP3+ regulatory T cells, and that these associations are particularly evident in luminal A tumours. Interestingly, FGFR2 expression did not correlate with the overall density of immune infiltrates but showed specificity for these key immunosuppressive cell subsets. This suggests that FGFR2 may influence (or be influenced by) the qualitative composition of the TIME rather than its overall magnitude.

Several studies have explored the immunological consequences of FGFR signalling, mainly in triple-negative breast cancer (TNBC), where FGFR activity has been linked to immune exclusion and resistance to immune checkpoint inhibitors (8, 10, 19, 20). The current work shifts the focus to luminal BCa, where the biological context in ER+ tumours differs substantially, both in terms of baseline immunogenicity and the role of FGFR2 in BCa signalling network. Here, we show that the immune effects of FGFR2 are detectable at the earliest stages of tumour development and appear to be restricted to the ER+ subtype. This supports the idea that the subtype-specific immune environment provides the setting favourable for the crosstalk between FGFR2-mediated and ER pathways to promote tumour evolution.

The subtype-specific nature of these correlations may also suggest that the immunomodulatory function of FGFR2 may depend on the functional ER signalling. This would align with previous observations that ER activation can modulate immune cell composition, including suppressing pro-inflammatory macrophage activity and influencing the accumulation of PD-1+ CD8+ T cells (21–23). In luminal tumours, FGFR2 and ER pathways may thus act synergistically to regulate selective immune cell recruitment and establish a microenvironment permissive to immune evasion. Moreover, the differential correlations observed only in luminal A tumours—despite similar levels of FGFR2 expression across subtypes—highlight the importance of hormonal context in modulating FGFR2-immune interactions.

Taken together, although the study is limited to a relatively small cohort, the findings suggest that FGFR2 expression may be associated with features of an immunosuppressive TIME in luminal BCa, and could serve as a potential biomarker for identifying this tumour microenvironment subtype. While the observed correlations between FGFR2 and CD163^+^ or FOXP3^+^ immune cells are modest, their selective presence in ER^+^ tumours is noteworthy and warrants further investigation. Importantly, the current data do not allow us to determine whether FGFR2 actively modulates the immune microenvironment or is itself regulated by it. Future studies will be necessary to elucidate the directionality of this interaction and to explore whether FGFR2 contributes to mechanisms of resistance to endocrine therapy. Prospective studies integrating transcriptomic and spatial profiling approaches will be instrumental in clarifying this crosstalk and may inform rational therapeutic strategies targeting both tumour-intrinsic FGFR2 signalling and the TIME.

## 5 Limitation of the study

A key limitation of this study is the relatively small sample size in certain subgroups, notably DCIS and non-luminal breast cancers, which reduces statistical power and may affect the robustness and generalizability of the observed associations. These findings should therefore be interpreted with caution and validated in larger, independent cohorts.

Another limitation is the absence of correction for multiple testing, which increases the risk of type I error. Given the exploratory nature of this study and the relatively small sample size, we chose to report unadjusted p-values, prioritizing biological plausibility and consistency across analyses. Nonetheless, these results should be interpreted as hypothesis-generating and validated in larger studies with appropriate statistical corrections.

## Data Availability

The original contributions presented in the study are included in the article/**Supplementary Material**. Further inquiries can be directed to the corresponding authors.
