# Supplementary material for: FGFR2 expression relates to subtype-specific tumour microenvironment (TIME) during luminal breast cancer evolution
Source: Front Oncol. 2025 Sep 12;15:1655438. doi: 10.3389/fonc.2025.1655438 (PMC12463615; doi:10.3389/fonc.2025.1655438)
Supplement: Supplementary file 1 [file Presentation1.pptx]

## Slide 1
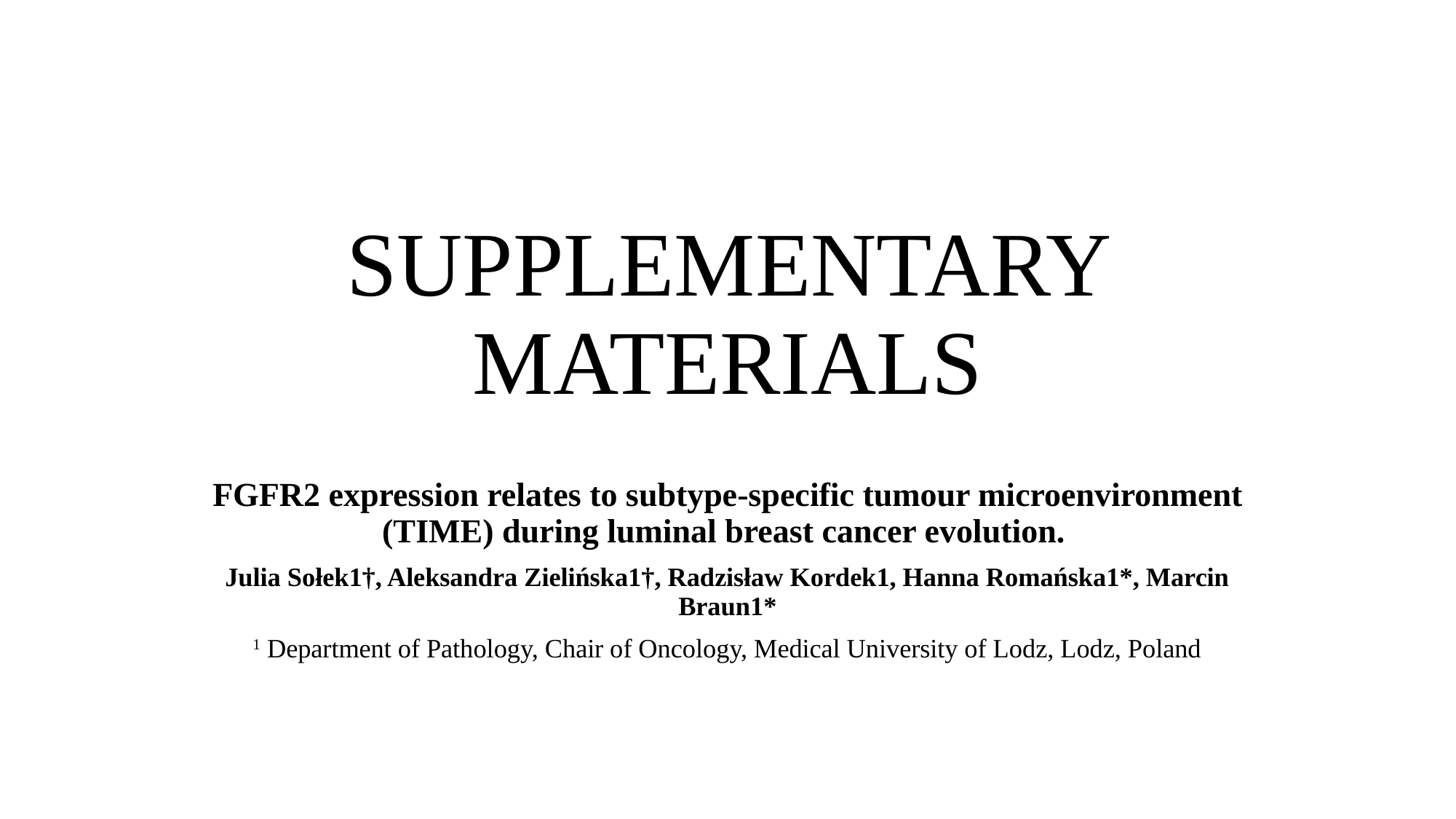

# SUPPLEMENTARY MATERIALS
FGFR2 expression relates to subtype-specific tumour microenvironment (TIME) during luminal breast cancer evolution.
Julia Sołek1†, Aleksandra Zielińska1†, Radzisław Kordek1, Hanna Romańska1*, Marcin Braun1*
1 Department of Pathology, Chair of Oncology, Medical University of Lodz, Lodz, Poland

## Slide 2
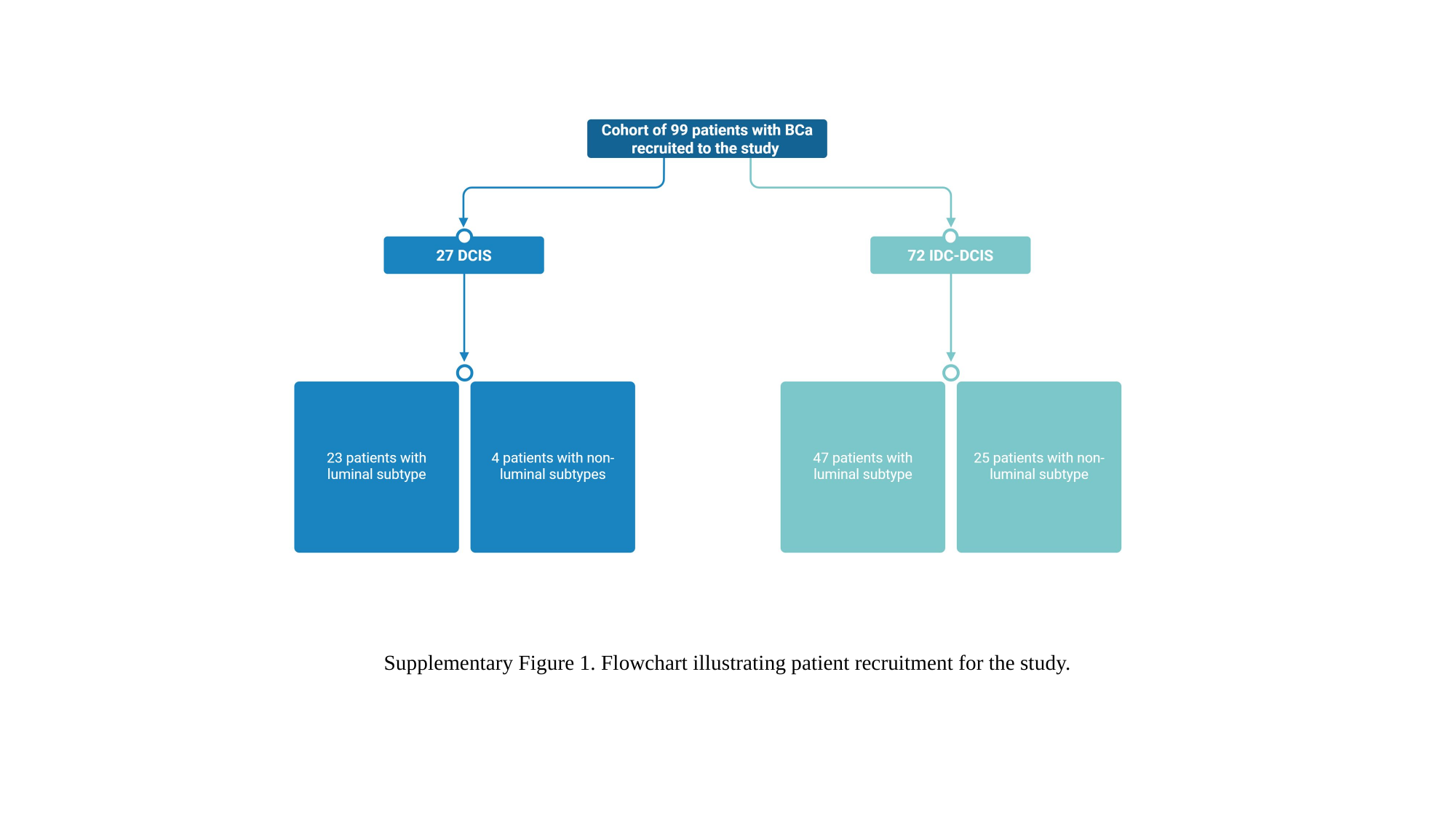

Supplementary Figure 1. Flowchart illustrating patient recruitment for the study.

## Slide 3
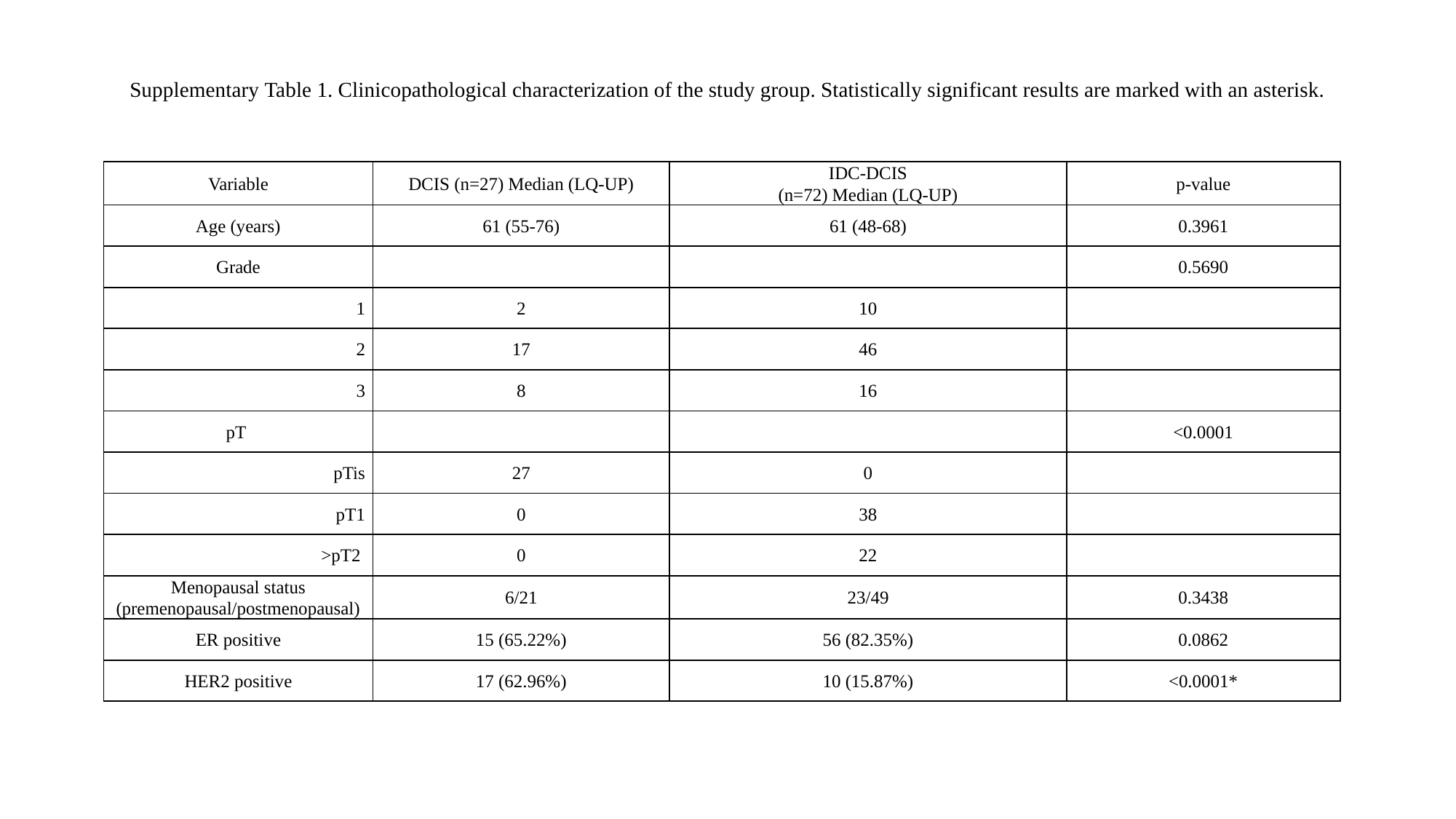

Supplementary Table 1. Clinicopathological characterization of the study group. Statistically significant results are marked with an asterisk.
| Variable | DCIS (n=27) Median (LQ-UP) | IDC-DCIS (n=72) Median (LQ-UP) | p-value |
| --- | --- | --- | --- |
| Age (years) | 61 (55-76) | 61 (48-68) | 0.3961 |
| Grade | | | 0.5690 |
| 1 | 2 | 10 | |
| 2 | 17 | 46 | |
| 3 | 8 | 16 | |
| pT | | | <0.0001 |
| pTis | 27 | 0 | |
| pT1 | 0 | 38 | |
| >pT2 | 0 | 22 | |
| Menopausal status (premenopausal/postmenopausal) | 6/21 | 23/49 | 0.3438 |
| ER positive | 15 (65.22%) | 56 (82.35%) | 0.0862 |
| HER2 positive | 17 (62.96%) | 10 (15.87%) | <0.0001\* |

## Slide 4
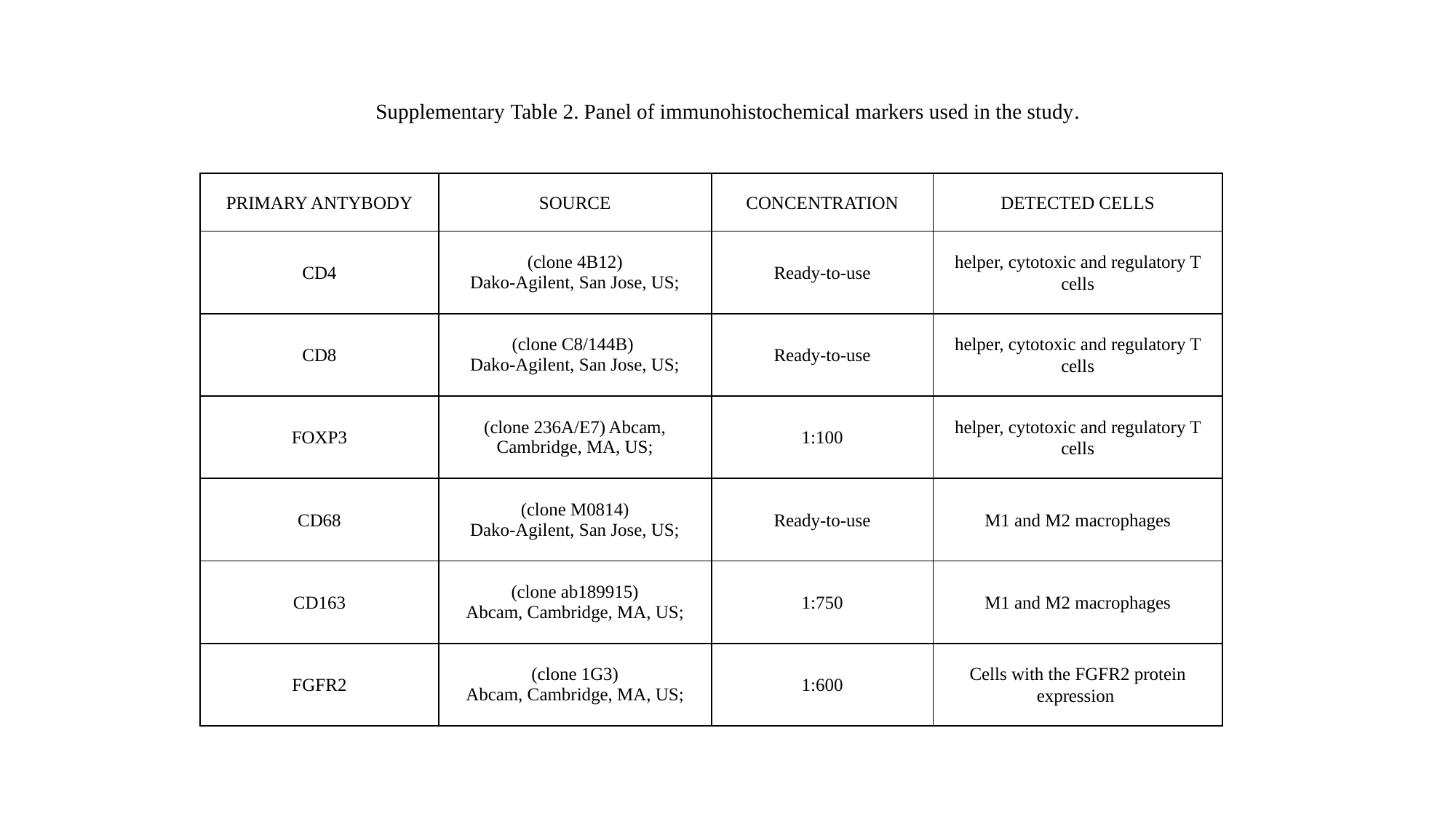

Supplementary Table 2. Panel of immunohistochemical markers used in the study.
| PRIMARY ANTYBODY | SOURCE | CONCENTRATION | DETECTED CELLS |
| --- | --- | --- | --- |
| CD4 | (clone 4B12) Dako-Agilent, San Jose, US; | Ready-to-use | helper, cytotoxic and regulatory T cells |
| CD8 | (clone C8/144B) Dako-Agilent, San Jose, US; | Ready-to-use | helper, cytotoxic and regulatory T cells |
| FOXP3 | (clone 236A/E7) Abcam, Cambridge, MA, US; | 1:100 | helper, cytotoxic and regulatory T cells |
| CD68 | (clone M0814) Dako-Agilent, San Jose, US; | Ready-to-use | M1 and M2 macrophages |
| CD163 | (clone ab189915) Abcam, Cambridge, MA, US; | 1:750 | M1 and M2 macrophages |
| FGFR2 | (clone 1G3) Abcam, Cambridge, MA, US; | 1:600 | Cells with the FGFR2 protein expression |

## Slide 5
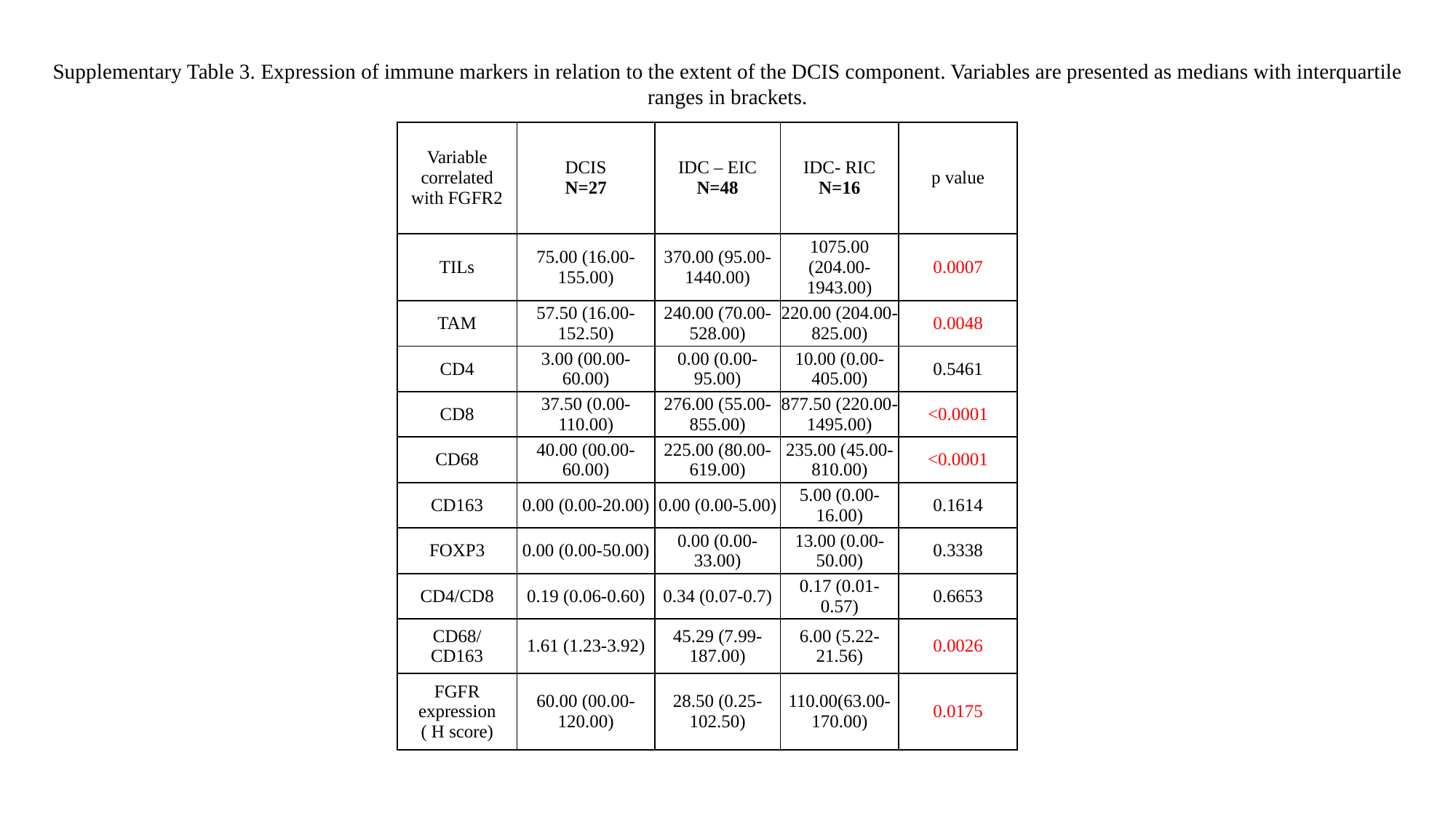

Supplementary Table 3. Expression of immune markers in relation to the extent of the DCIS component. Variables are presented as medians with interquartile ranges in brackets.
| Variable correlated with FGFR2 | DCIS N=27 | IDC – EIC N=48 | IDC- RIC N=16 | p value |
| --- | --- | --- | --- | --- |
| TILs | 75.00 (16.00-155.00) | 370.00 (95.00-1440.00) | 1075.00 (204.00-1943.00) | 0.0007 |
| TAM | 57.50 (16.00-152.50) | 240.00 (70.00-528.00) | 220.00 (204.00-825.00) | 0.0048 |
| CD4 | 3.00 (00.00-60.00) | 0.00 (0.00-95.00) | 10.00 (0.00-405.00) | 0.5461 |
| CD8 | 37.50 (0.00-110.00) | 276.00 (55.00-855.00) | 877.50 (220.00-1495.00) | <0.0001 |
| CD68 | 40.00 (00.00-60.00) | 225.00 (80.00-619.00) | 235.00 (45.00-810.00) | <0.0001 |
| CD163 | 0.00 (0.00-20.00) | 0.00 (0.00-5.00) | 5.00 (0.00-16.00) | 0.1614 |
| FOXP3 | 0.00 (0.00-50.00) | 0.00 (0.00-33.00) | 13.00 (0.00-50.00) | 0.3338 |
| CD4/CD8 | 0.19 (0.06-0.60) | 0.34 (0.07-0.7) | 0.17 (0.01-0.57) | 0.6653 |
| CD68/CD163 | 1.61 (1.23-3.92) | 45.29 (7.99-187.00) | 6.00 (5.22-21.56) | 0.0026 |
| FGFR expression ( H score) | 60.00 (00.00-120.00) | 28.50 (0.25-102.50) | 110.00(63.00-170.00) | 0.0175 |

## Slide 6
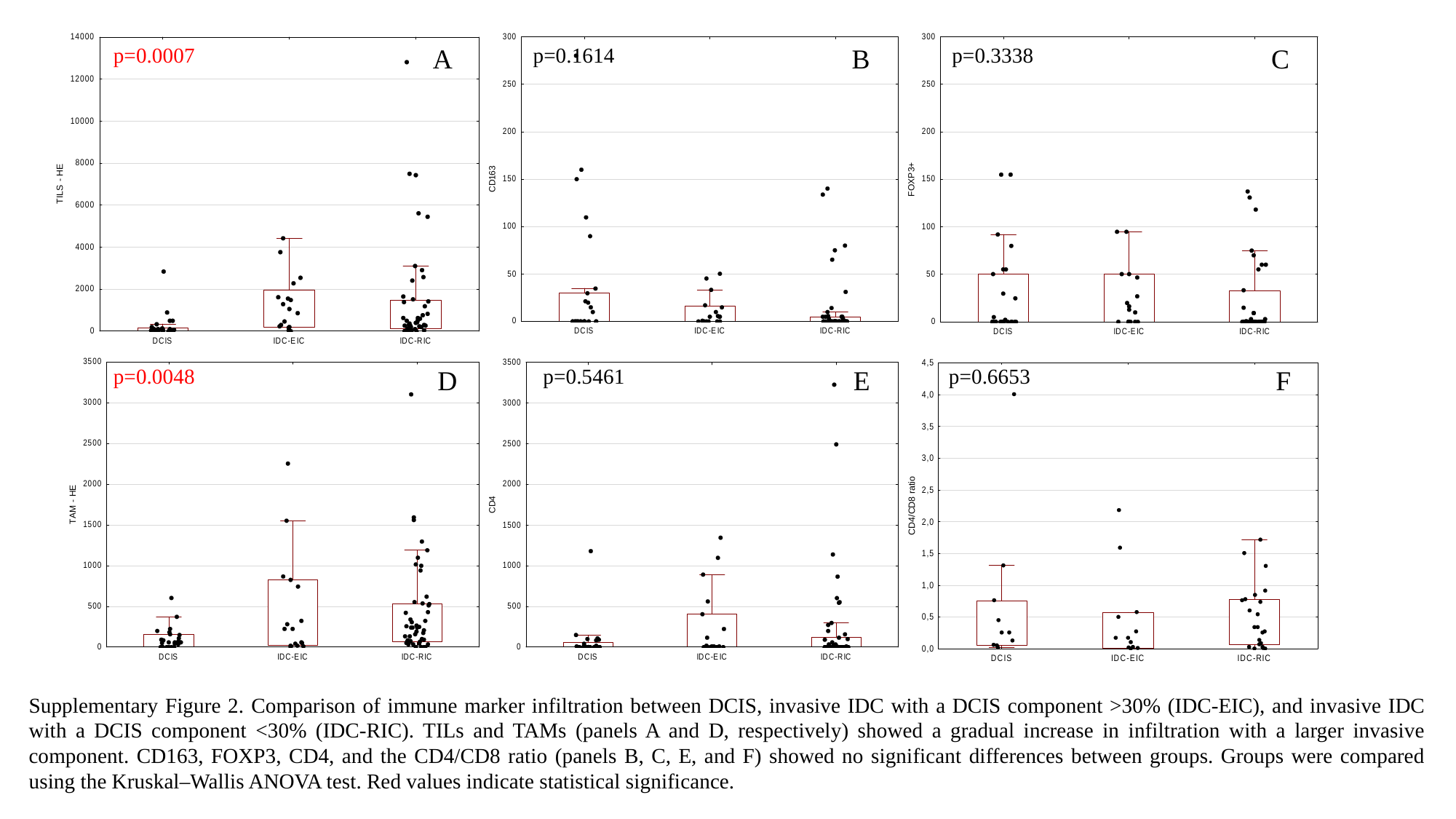

p=0.0007
A
p=0.1614
B
p=0.3338
C
p=0.0048
D
p=0.5461
E
p=0.6653
F
Supplementary Figure 2. Comparison of immune marker infiltration between DCIS, invasive IDC with a DCIS component >30% (IDC-EIC), and invasive IDC with a DCIS component <30% (IDC-RIC). TILs and TAMs (panels A and D, respectively) showed a gradual increase in infiltration with a larger invasive component. CD163, FOXP3, CD4, and the CD4/CD8 ratio (panels B, C, E, and F) showed no significant differences between groups. Groups were compared using the Kruskal–Wallis ANOVA test. Red values indicate statistical significance.

## Slide 7
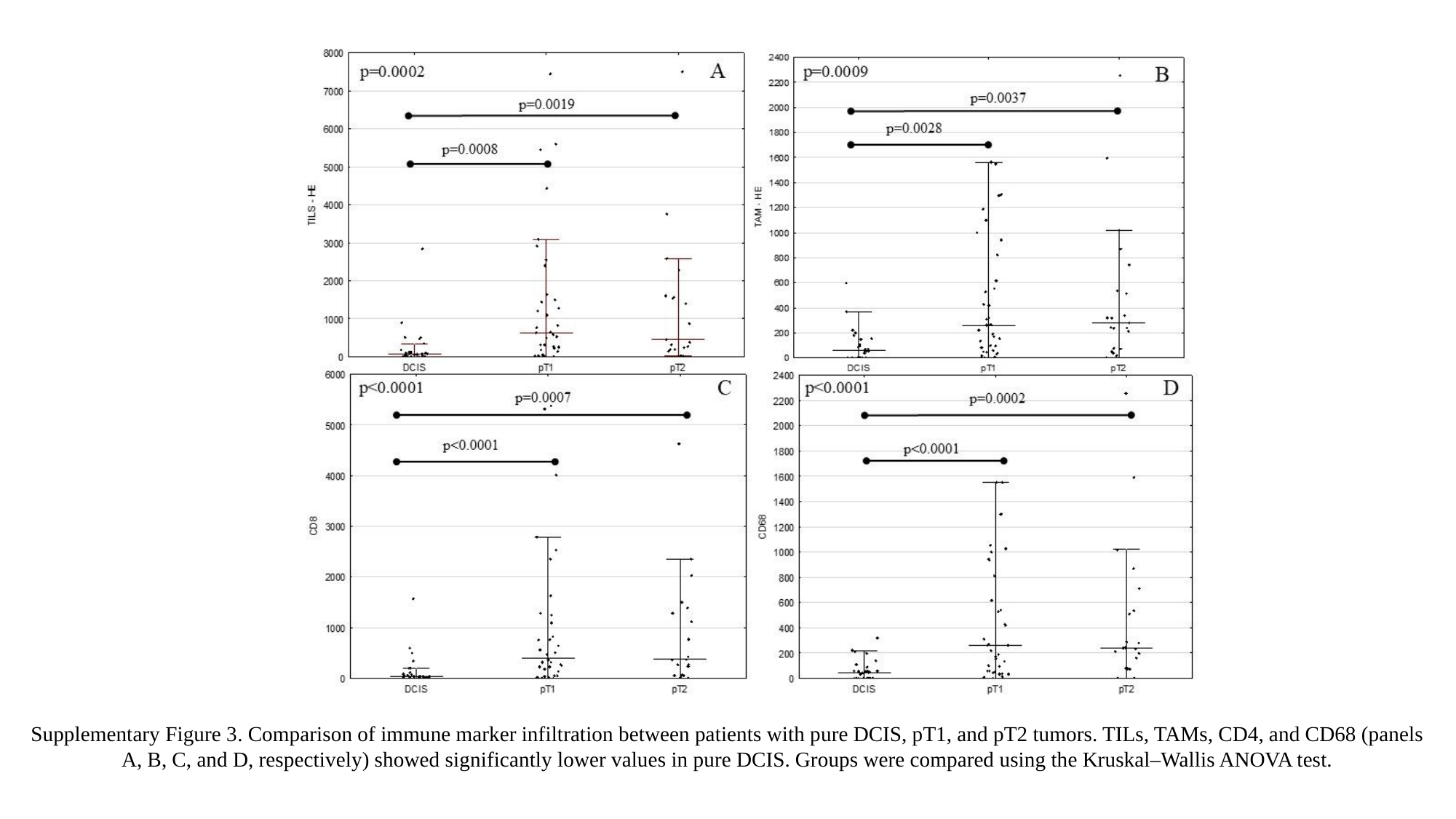

Supplementary Figure 3. Comparison of immune marker infiltration between patients with pure DCIS, pT1, and pT2 tumors. TILs, TAMs, CD4, and CD68 (panels A, B, C, and D, respectively) showed significantly lower values in pure DCIS. Groups were compared using the Kruskal–Wallis ANOVA test.

## Slide 8
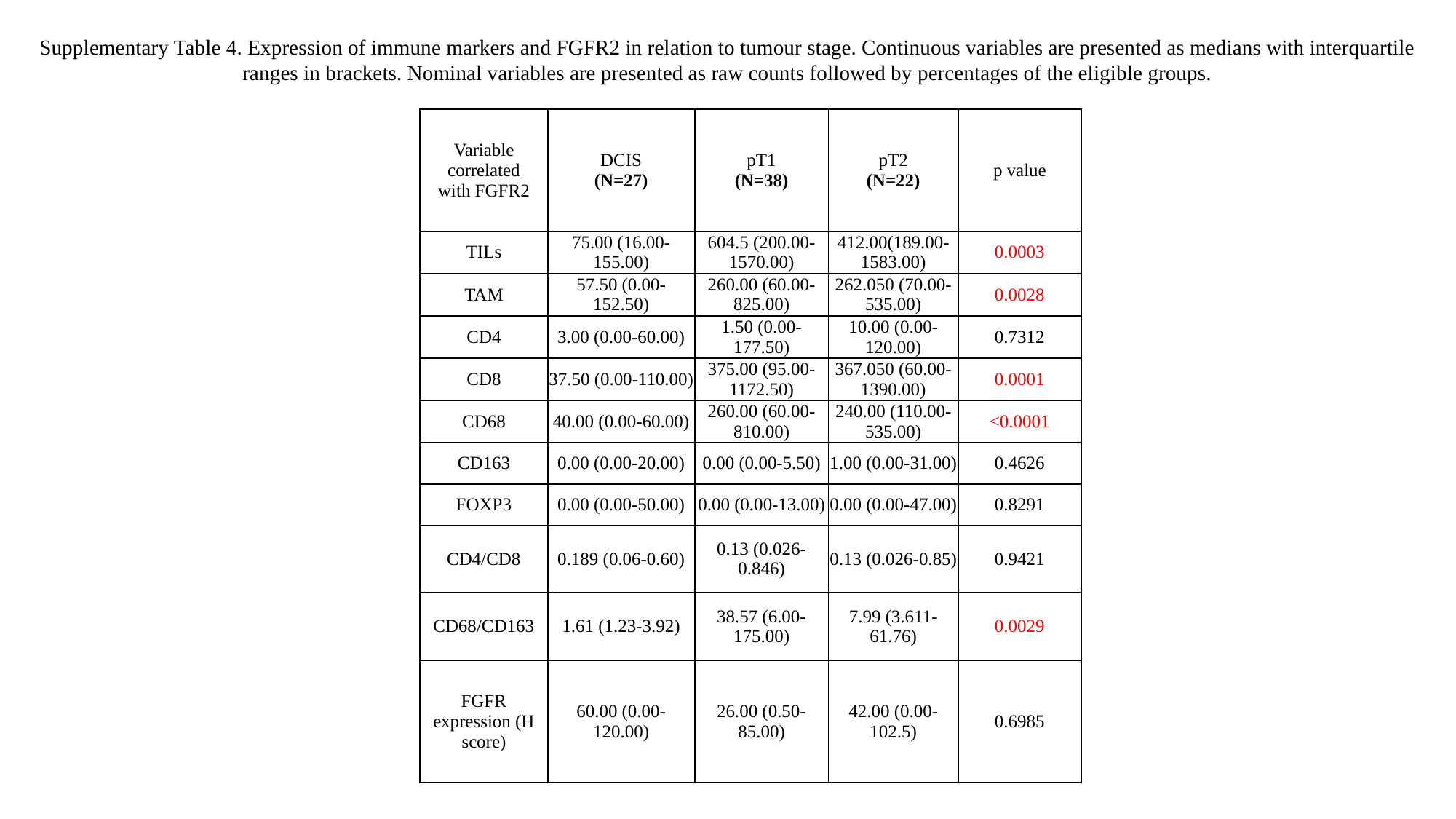

Supplementary Table 4. Expression of immune markers and FGFR2 in relation to tumour stage. Continuous variables are presented as medians with interquartile ranges in brackets. Nominal variables are presented as raw counts followed by percentages of the eligible groups.
| Variable correlated with FGFR2 | DCIS (N=27) | pT1 (N=38) | pT2 (N=22) | p value |
| --- | --- | --- | --- | --- |
| TILs | 75.00 (16.00-155.00) | 604.5 (200.00-1570.00) | 412.00(189.00-1583.00) | 0.0003 |
| TAM | 57.50 (0.00-152.50) | 260.00 (60.00-825.00) | 262.050 (70.00-535.00) | 0.0028 |
| CD4 | 3.00 (0.00-60.00) | 1.50 (0.00-177.50) | 10.00 (0.00-120.00) | 0.7312 |
| CD8 | 37.50 (0.00-110.00) | 375.00 (95.00-1172.50) | 367.050 (60.00-1390.00) | 0.0001 |
| CD68 | 40.00 (0.00-60.00) | 260.00 (60.00-810.00) | 240.00 (110.00-535.00) | <0.0001 |
| CD163 | 0.00 (0.00-20.00) | 0.00 (0.00-5.50) | 1.00 (0.00-31.00) | 0.4626 |
| FOXP3 | 0.00 (0.00-50.00) | 0.00 (0.00-13.00) | 0.00 (0.00-47.00) | 0.8291 |
| CD4/CD8 | 0.189 (0.06-0.60) | 0.13 (0.026-0.846) | 0.13 (0.026-0.85) | 0.9421 |
| CD68/CD163 | 1.61 (1.23-3.92) | 38.57 (6.00-175.00) | 7.99 (3.611-61.76) | 0.0029 |
| FGFR expression (H score) | 60.00 (0.00-120.00) | 26.00 (0.50-85.00) | 42.00 (0.00-102.5) | 0.6985 |

## Slide 9
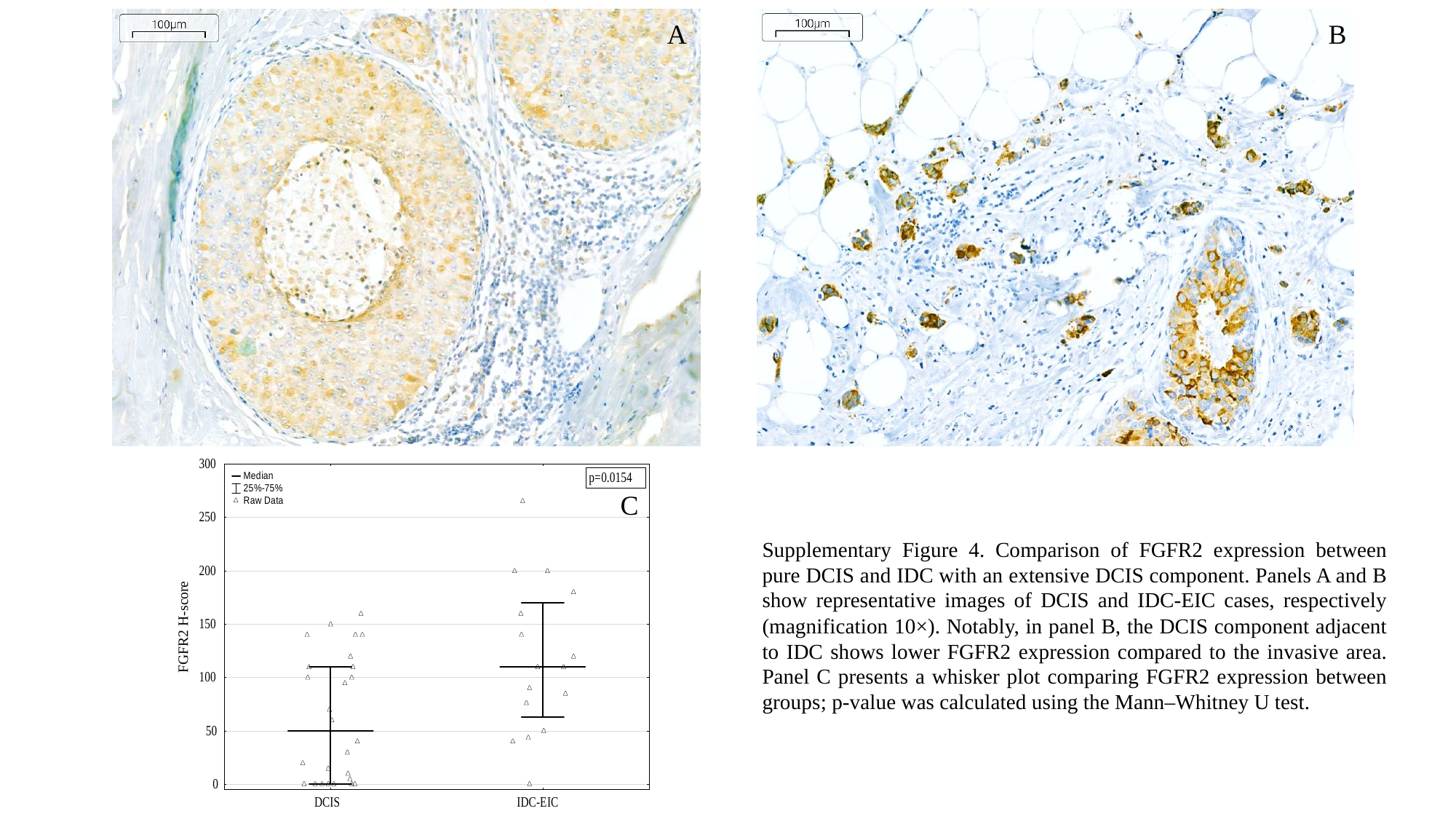

A
B
C
Supplementary Figure 4. Comparison of FGFR2 expression between pure DCIS and IDC with an extensive DCIS component. Panels A and B show representative images of DCIS and IDC-EIC cases, respectively (magnification 10×). Notably, in panel B, the DCIS component adjacent to IDC shows lower FGFR2 expression compared to the invasive area. Panel C presents a whisker plot comparing FGFR2 expression between groups; p-value was calculated using the Mann–Whitney U test.

## Slide 10
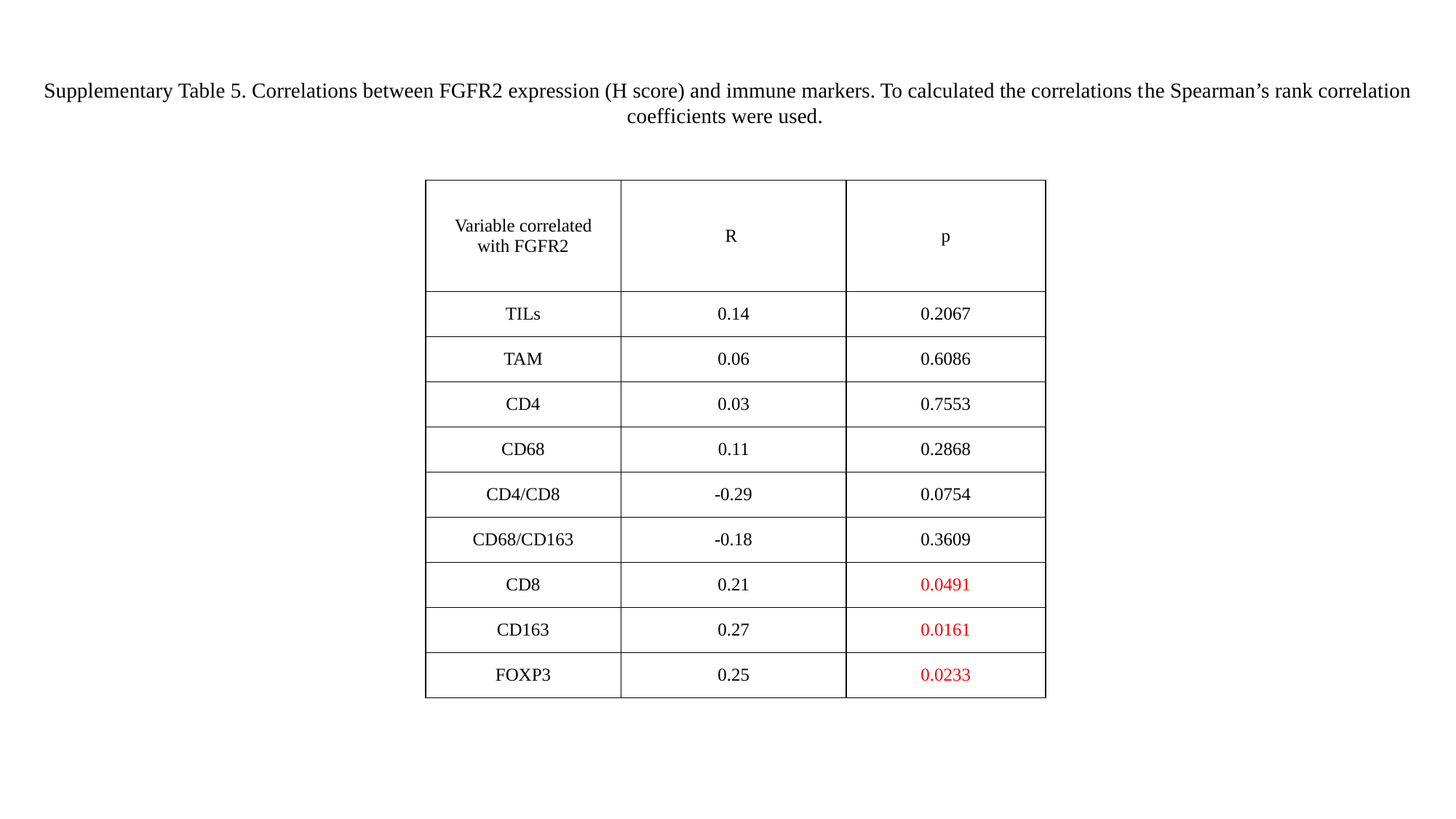

Supplementary Table 5. Correlations between FGFR2 expression (H score) and immune markers. To calculated the correlations the Spearman’s rank correlation coefficients were used.
| Variable correlated with FGFR2 | R | p |
| --- | --- | --- |
| TILs | 0.14 | 0.2067 |
| TAM | 0.06 | 0.6086 |
| CD4 | 0.03 | 0.7553 |
| CD68 | 0.11 | 0.2868 |
| CD4/CD8 | -0.29 | 0.0754 |
| CD68/CD163 | -0.18 | 0.3609 |
| CD8 | 0.21 | 0.0491 |
| CD163 | 0.27 | 0.0161 |
| FOXP3 | 0.25 | 0.0233 |

## Slide 11
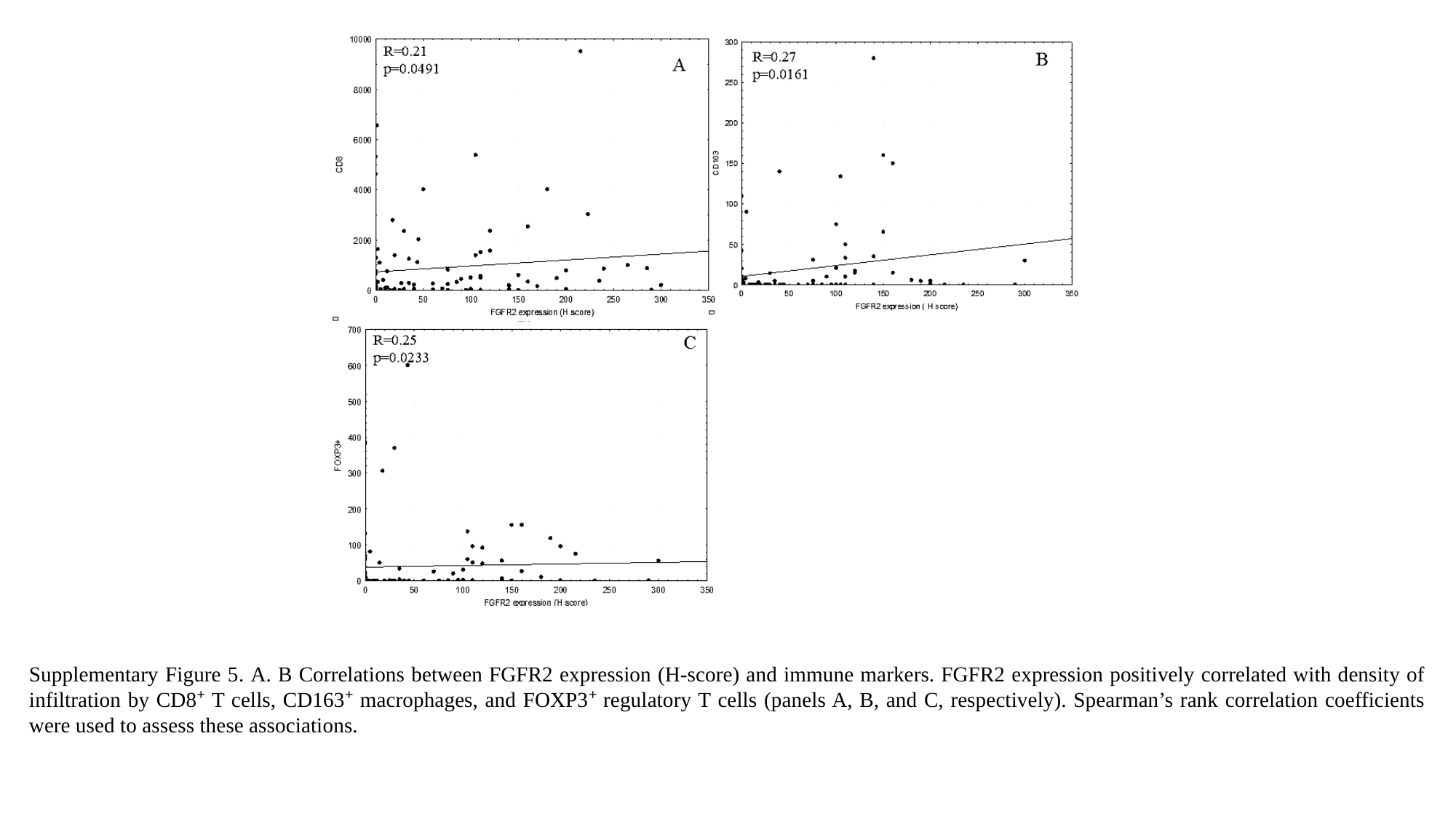

Supplementary Figure 5. A. B Correlations between FGFR2 expression (H-score) and immune markers. FGFR2 expression positively correlated with density of infiltration by CD8⁺ T cells, CD163⁺ macrophages, and FOXP3⁺ regulatory T cells (panels A, B, and C, respectively). Spearman’s rank correlation coefficients were used to assess these associations.

## Slide 12
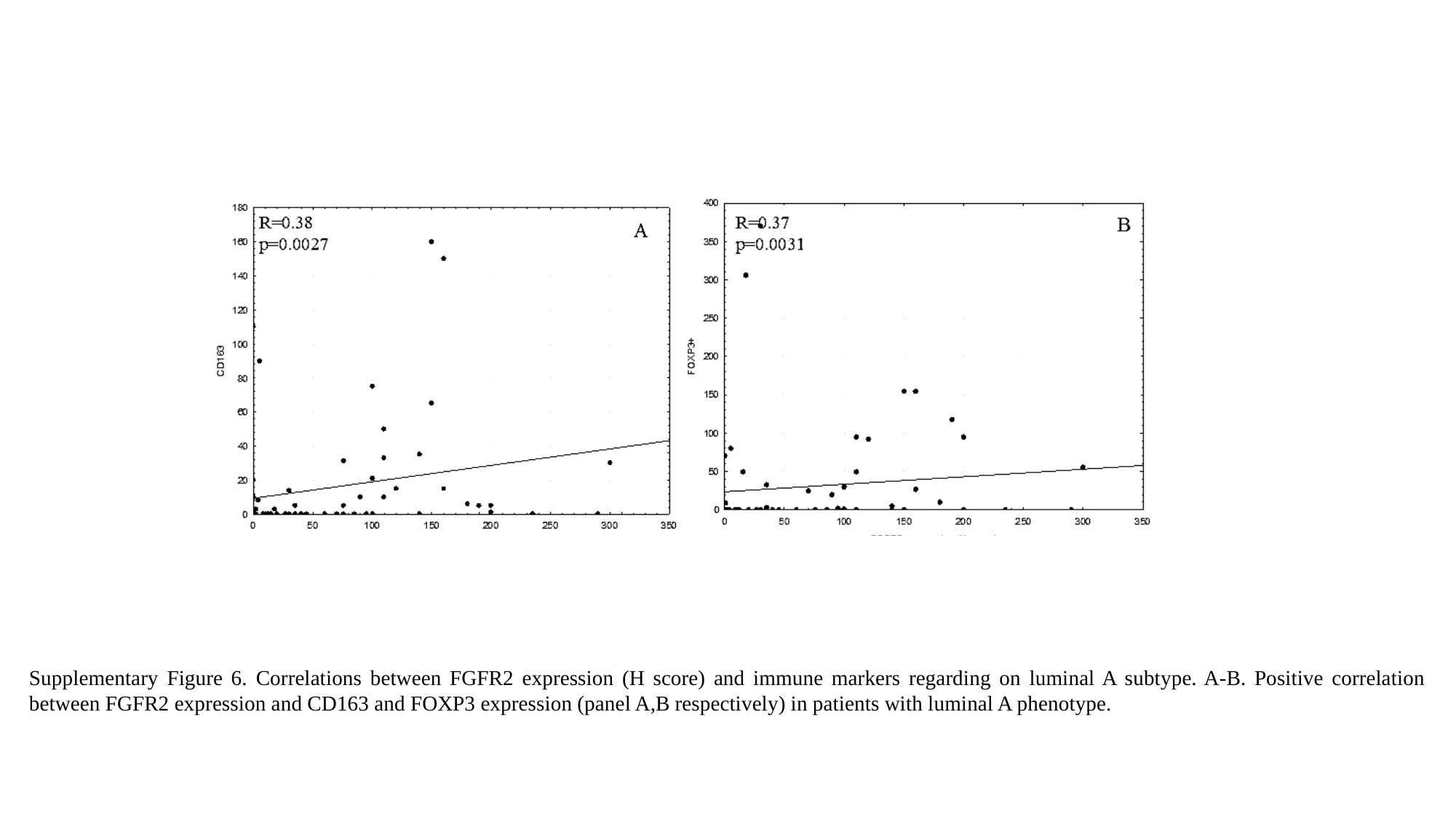

Supplementary Figure 6. Correlations between FGFR2 expression (H score) and immune markers regarding on luminal A subtype. A-B. Positive correlation between FGFR2 expression and CD163 and FOXP3 expression (panel A,B respectively) in patients with luminal A phenotype.
